# Occlusal Plane, Mandibular Position and Dentoalveolar Changes during the Orthodontic Treatment with the Use of Mini-Screws

**DOI:** 10.3390/dj12090278

**Published:** 2024-08-30

**Authors:** Julián David Gómez-Bedoya, Pablo Arley Escobar-Serna, Eliana Midori Tanaka-Lozano, Andrés A. Agudelo-Suárez, Diana Milena Ramírez-Ossa

**Affiliations:** 1 Faculty of Dentistry, University of Antioquia, Medellin 050010, Colombia; julian.gomez4@udea.edu.co (J.D.G.-B.); pablo.escobar@udea.edu.co (P.A.E.-S.); 2 Faculty of Dentistry, University “El Bosque”, Bogota 110121, Colombia; emtanaka@gmail.com

**Keywords:** dental occlusion, jaw relation record, orthodontic anchorage procedures, orthodontic appliances

## Abstract

This study aimed to describe the changes produced on the occlusal plane (OP), the mandibular position and the dentoalveolar compensations of patients with distalization of the maxillary/mandibular arch assisted by mini-screws (MS). A descriptive case–series study was performed using the digital lateral cephalograms (DLC) of nine patients who underwent orthodontic treatment and required the use of MS for a complete distalization of the maxillary/mandibular arch. Records were collected at three different times (T1–T2–T3) and digitally analyzed (variables: Skeletal diagnosis; maxillary occlusal plane; position of the maxilla/mandible; and dentoalveolar changes of the distalization arch tracing the longitudinal axis of incisors/molars regarding the palatal/mandibular plane). Findings show that the OP varied from T1–T2–T3 in all cases, indicating its stepping or flattening. ODI, APDI, SNA, SNB, and ANB changed minimally in all cases, without variations in the mandibular position or in the skeletal diagnosis. Dentoalveolar measurements also showed differences between T1–T2–T3. In summary, conventional orthodontic treatment modified the OP during the first phase of treatment. Moreover, the distalization mechanics with MS changed the OP and produced dentoalveolar changes, mainly in the inclination of incisors and molars. Other measures considered in the study did not change substantially.

## 1. Introduction

The inclination of the occlusal plane (OP) is an important factor in determining craniofacial growth, where the position of the maxilla determines the upper OP, which influences the position and function of the mandible [1,2]. Tanaka and Sato described the OP as a line drawn from the incisal edge of the maxillary central incisor to the midpoint of the maxillary first molar on the occlusal surface [3]. It can be divided into anterior OP (AOP) and posterior OP (POP). The AOP runs from the incisal edge of the maxillary central incisor to the cusp tip of the maxillary second premolar, and the POP runs from the cusp tip of the maxillary second premolar to the midpoint of the maxillary second molar or third molar (if erupted) on the occlusal surface [3]. A patient with mesioclusion has an excessive maxillary vertical dimension, with a subsequent flattening of the OP; the mandible moves anteriorly, protrudes, and increases secondary condylar growth [1]. In a patient with distoclusion, the maxilla has greater anteroposterior growth, and the vertical deficiency leads to the tipping of the OP; the mandible adapts by rotating posteriorly, which obstructs condylar growth [1]. This shows the important role the OP plays in the development of malocclusion because correcting its inclination, the dental interdigitation and the position of the jaw and the condyle will improve the patient’s function [1].

Since the popularization of mini-screws (MS) used as skeletal anchorage in orthodontic treatment in the late 1990s [4], the way of controlling anchorage has changed. As protocols have been perfected, their use has been expanded, not only for anchorage but also for the development of new orthodontic biomechanics [5,6]. This is how today they are a tool that helps make different dental movements easier and more efficient, such as incisor retrusion, posterior intrusion, closure of spaces, impacted teeth traction and management of Class II or Class III with distalization of complete arches [5]. However, the mechanics implemented with MS are not clear regarding the behavior of the OP; if a complete distalization of the arch is performed, dentoalveolar compensations could be present, such as molar intrusion and verticalization, the inclination of incisors, dentoalveolar expansion among other movements, [7] and, depending on the site of application of the force and the device placement, the OP would change both vertically and anteroposteriorly, possibly altering the position of the mandible [7,8].

To the best of the authors’ knowledge, the scientific evidence of this management is still poor, with gaps regarding the behavior of OP with MS in cases of complete distalization of the upper and/or lower arch from the radiographic point of view. Therefore, the aim of this study is to describe the changes produced in the OP, the mandibular position and dentoalveolar compensations of patients with orthodontic treatment in which MS was selected as a biomechanical tool for the distalization of the maxillary or mandibular arch.

## 2. Materials and Methods

### 2.1. Design, Data Collection Techniques and Study Variables

A descriptive case series study was performed through the report of digital lateral cephalograms (DLC) records of routine patients who underwent orthodontic treatment and required the use of MS as a biomechanical tool for a complete distalization of the maxillary and/or mandibular arch. It is important to highlight that their orthodontic purpose was not the management of the OP but the distalization of dental arches, for example, to avoid dental extractions. All the patients were chosen by convenience from the graduate program of orthodontics at the University of Antioquia and the private practice of one orthodontist in the city of Medellín, Colombia.

**Figure 1:**
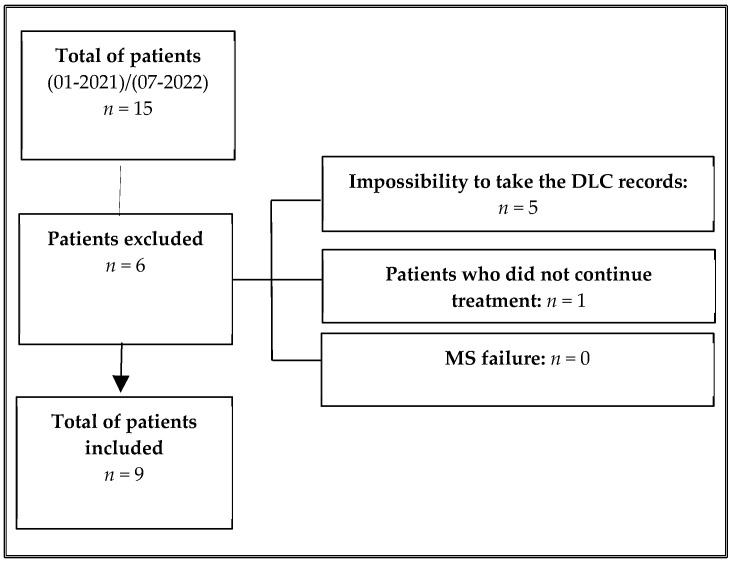
Selection process of patients.

Patients and their records were collected over a period of 18 months from January 2021 to July 2022 and were pre-selected according to the following inclusion criteria: (1) patients of both sexes who lived in Colombia; (2) initial DLC records available; (3) use of MS as a biomechanical anchorage method for distalization of the dentition in the maxillary or mandibular arch; (4) vertebral maturation stage greater than 6 in the DLC according to Baccetti’s analysis at the moment of MS installation [9]; (5) no supernumerary teeth or maxillary lesions observed in the panoramic radiograph; and (6) patients who did not report systemic diseases or syndromes. Patients with the following findings were excluded: (1) impossibility of taking the DLC records after the MS procedure; (2) patients who refused to continue the treatment with MS; and (3) MS failure that cannot be relocated in a short period of time (no more than 2 weeks). Records of 15 patients were collected, but only 9 patients were included (Figure 1).

In addition to the initial DLC records (T1), two others were taken (T2, T3): T1 corresponded to the beginning of the treatment, where the OP was analyzed prior to any orthodontic intervention and the skeletal sagittal and vertical diagnosis were determined; T2 corresponded to the DLC taken during the 48 h after the installation of MS, where the changes of the OP were analyzed prior to the use of the MS; and T3 corresponded to the DLC taken during the 48 h after stopping the MS distalization process, where the changes in OP due to the use of the MS as part of the biomechanical strategy were analyzed. There was no distinction with respect to the radiological center because the analysis corresponds to angular measurements, which are reliable and independent of the radiological equipment [10].

**Figure 2:**
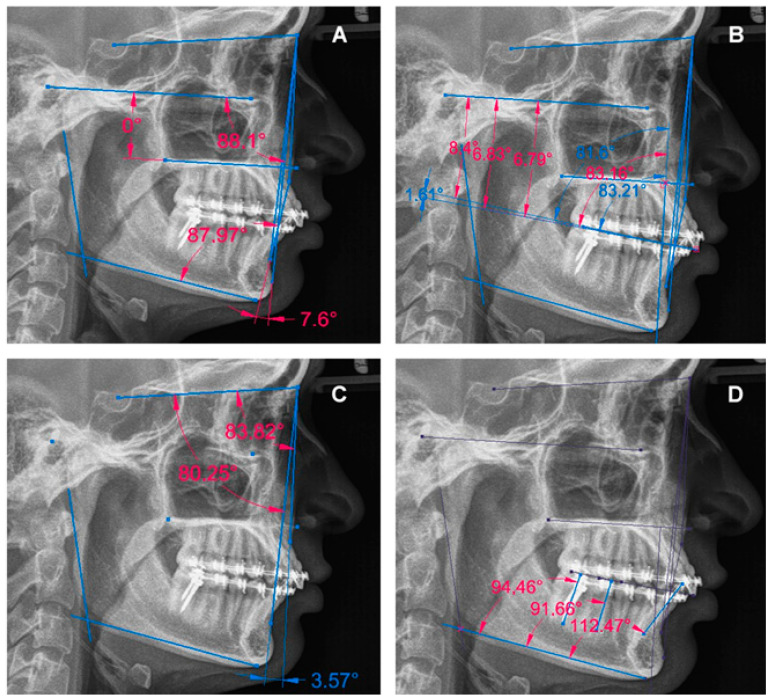
Digital lateral cephalogram analysis example. (**A**) Skeletal diagnosis by Kim. (**B**) Occlusal plane by Tanaka and Sato. (**C**) Position of maxilla and mandible by Riedel. (**D**) Dentoalveolar changes analysis by the authors.

All radiographs were analyzed with the Solid Edge 2020 Academic Edition Siemens© PLM software. Measurements were made according to the following criteria: Skeletal diagnosis using the overbite depth indicator (ODI) and the anteroposterior dysplasia indicator (APDI) as described by Kim [11,12]; maxillary occlusal plane according to Tanaka and Sato analysis [3]; position of the maxilla and mandible with Riedel analysis [13]; and dentoalveolar changes of incisors and molars of the distalization arch tracing their longitudinal axis regarding the palatal plane or the mandibular plane according to the authors (Supplementary Table S1, Figure 2). The data corresponding to the sociodemographic and orthodontic treatment variables were obtained directly from the clinical history in the final phase corresponding to the time of withdrawal of the MS or phase T3.

### 2.2. Bias Control

For the analysis of the radiographs, a theoretical calibration was performed in the first instance with two experts (D.M.R.-O and E.M.T.-L.). Also, the risk of bias was reduced, considering that the data provided by the orthodontist were recorded in the clinical history of each patient included in the research. Next, to assess reproducibility, the research group used Dahlberg’s method. This is a frequent measure for assessing random errors in cephalometric studies. The study had a small sample size (*n* = 9); by means of a pilot test with 5 cephalic radiographs, we found values ≅ 1.0. Although one of the difficulties in interpreting the size of the error is that there is almost no reference for an acceptable range, considering the clinical implications, the values could be acceptable for the study purposes [14].

### 2.3. Statistical and Other Analyses

According to the study purposes, we conducted a descriptive analysis of the variables included in the study. For some measures, we conducted Chi-square tests to observe statistically significant differences in the cephalometric results in T1, T2, and T3. For that means, we conducted normality tests and we decided to carry out non-parametric tests. Specifically, we used the Friedman test (Similar to the parametric repeated measures ANOVA) to detect differences in treatments across multiple test attempts. Finally, we conducted a qualitative description for each case, considering their variability, the particularities of the case report, and the clinical implications for orthodontics practice.

### 2.4. Ethical Considerations

This project was approved by the Committee for the Development of Research as well as the Ethics Committee of the Faculty of Dentistry (Institutional Review Board IRB 57-2020). None of the patients were specifically recruited for research purposes since all patients were routine orthodontic consultations; however, some of the records were taken for research purposes only, with the previous consent of the patients.

## 3. Results

### 3.1. General Characteristics of the Sample

Clinical and biomechanical variables are summarized in Supplementary Table S2. All patients were women (100%, *n* = 9) with a mean age of 23.33 years. The sagittal skeletal diagnosis was Class I in 22.2% of the cases (*n* = 2), Class II in 33.3% (*n* = 3), and Class III in 44.5% (*n* = 4); with regards to vertical relations, 22.2% patients were neutral angle (*n* = 2), 44.5% high angle (*n* = 4) and 33.3% low angle (*n* = 3). Dental relationships varied between Class I, Class II and Class III. Concerning the presence/absence of third molars in the distalization arch, 33.3% of the patients did not report their presence at the beginning of treatment (*n* = 3), 44.5% were referred to their extraction between T1–T2 (*n* = 4), and 22.2% of the patients kept them during all the study time (*n* = 2).

All patients used stainless steel (***SS***) appliances, and Roth prescription was the most used (77.7%, *n* = 7), being slot size 0.022″ × 0.028″ the preferred by the clinicians (88.8%, *n* = 8). Three main reasons were observed for choosing the distalization: to verticalize incisors (66.55%, *n* = 6), to achieve Class I canine relationship (22.3%, *n* = 2), and to increase sagittal discrepancy in a surgical case (11.15%, *n* = 1). The biomechanics used in the maxilla (33.4%, *n* = 3) or in the mandible (66.6%, *n* = 6) were diverse in terms of the archwires, but all the cases used elastic chain from an extra-alveolar ***MS*** to the arch or to a hook mesial to canines or premolars. Two patients underwent distalization of the opposite arch to improve their overjet after completing the first phase of MS distalization, but OP analysis was only performed on the main arch, where greater biomechanics were implemented.

**Figure 3:**
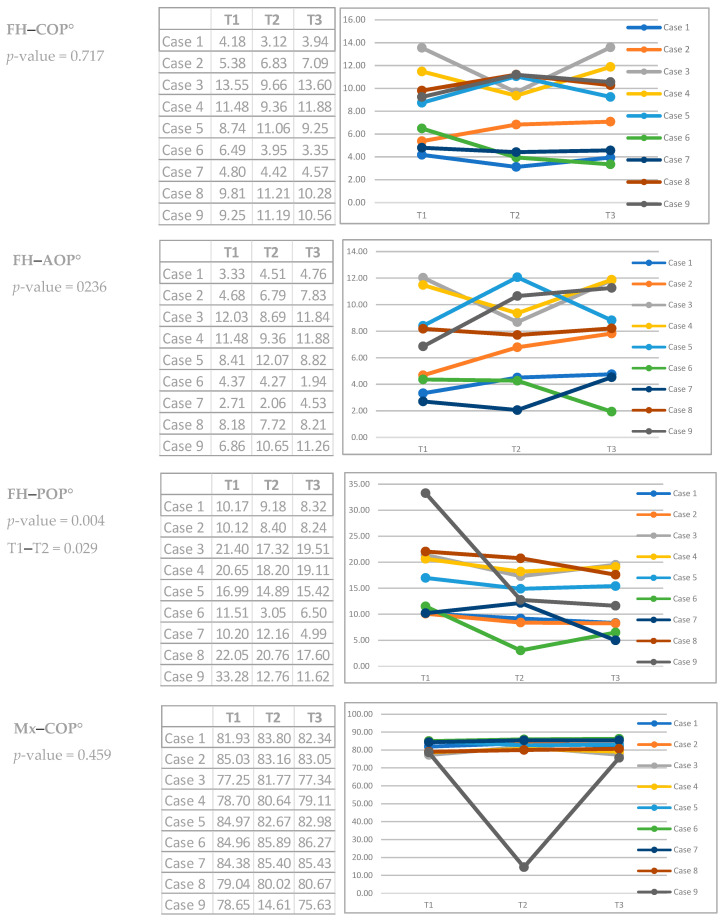

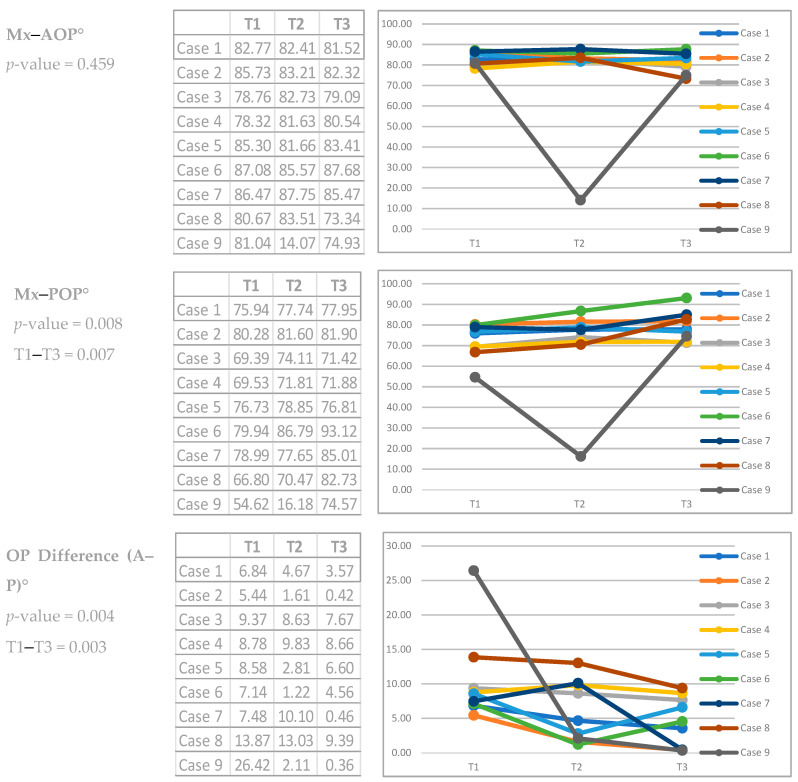
Cephalometric analysis results in T1, T2 and T3 (Occlusal plane). * The Chi-squared test was calculated by means of the Friedman test (Similar to the parametric repeated measures ANOVA) to detect differences in treatments across multiple test attempts. ** Case 3 was the only case that changed its sagittal skeletal diagnosis from Class II to Class I, according to Kim analysis.

**Figure 4:**
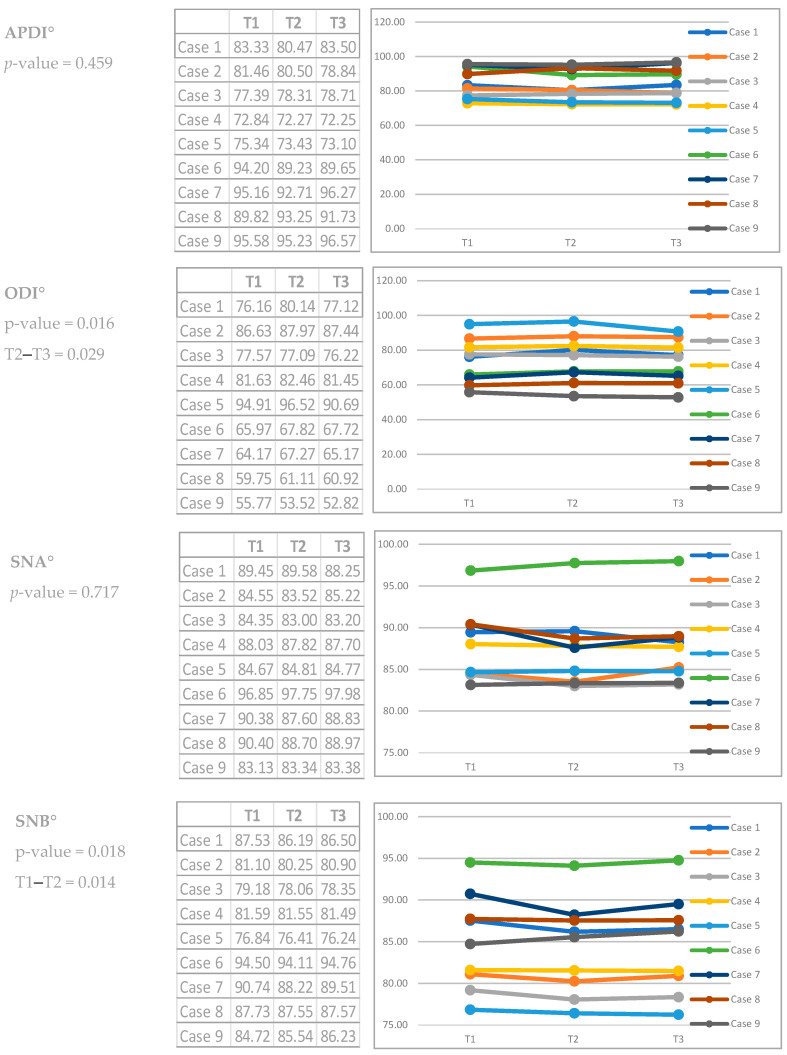

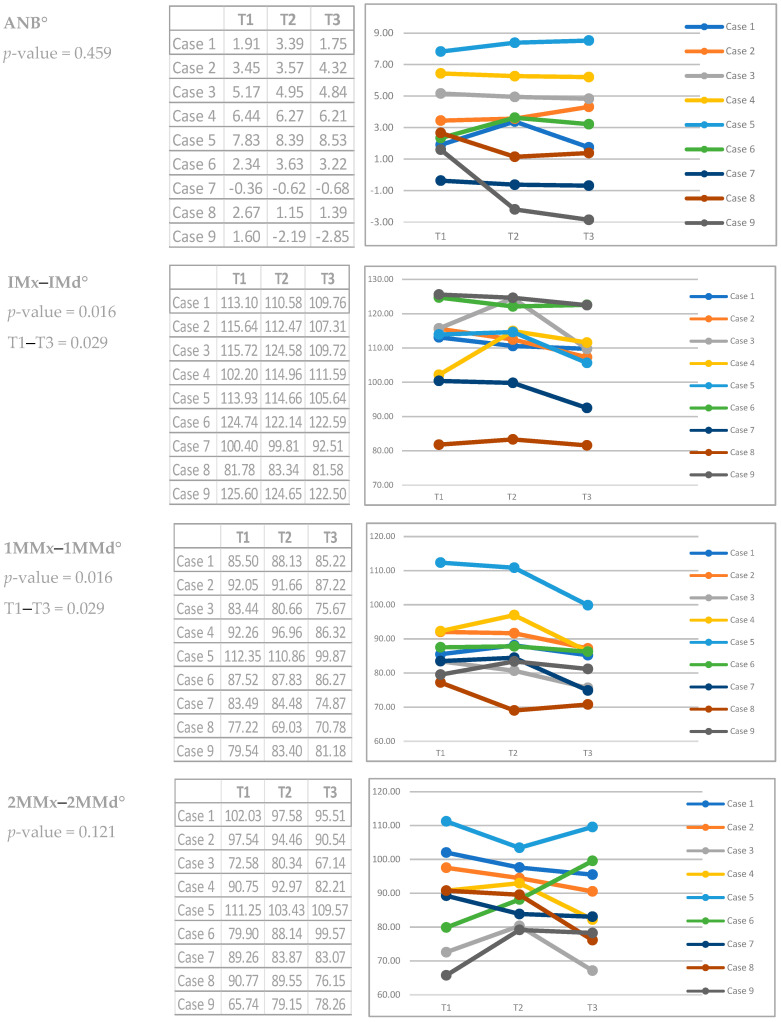
Cephalometric analysis results in T1, T2 and T3 (Skeletal and dentoalveolar relations). * The Chi-squared test was calculated by means of the Friedman test (Similar to the parametric repeated measures ANOVA) to detect differences in treatments across multiple test attempts. ** Case 3 was the only case that changed its sagittal skeletal diagnosis from Class II to Class I, according to Kim analysis.

The conventional occlusal plane (COP), the anterior occlusal plane (AOP) and the posterior occlusal plane (POP) varied from T1–T2–T3 in all cases; the values increased or decreased, indicating stepping or flattening of the OP (Supplementary Table S2 and Figure 3 and Figure 4). Dentoalveolar measurements (DMs) also show differences between T1–T2–T3. ODI, APDI, SNA, SNB and ANB measurements change minimally three times in all cases, without variations in the mandibular position or in the skeletal diagnosis, except in case 3 (Supplementary Table S2).

### 3.2. Report of the Specific Characteristics of the Cases

#### 3.2.1. Case 1

**Figure 5:**
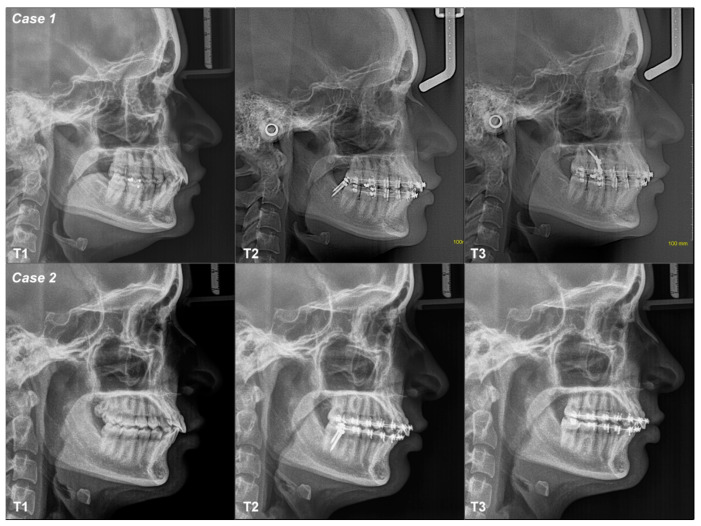
Class I patients digital lateral cephalograms.

A 29-year-old female patient with Class II canine relationships and skeletal Class I neutral angle. Her objective was to verticalize their lower incisors with two MS at the buccal shelf (BS), which was achieved after two months. The biomechanics included Roth 0.022″ × 0.028″ fixed appliances with a 0.018″ × 0.018″ SS continuous archwire with a force of 200–300 grs applied through a chain mesial to 34 and 44, above the center of resistance. The COP flattened from T1–T2 but tipped between T2–T3; AOP tipped between T1–T2–T3; POP flattened between T1–T2–T3. All DMs decreased, except for the mandibular first molar between T1–T2, which shows a mesial tip that decreased between T2–T3 (Figure 3, Figure 4 and Figure 5; Table 1).

#### 3.2.2. Case 2

A 20-year-old female patient with Class II canine relationships and skeletal Class I low angle. Her objective was to verticalize the incisors with two MS at the BS, achieved after four months. The biomechanics included Roth 0.022″ × 0.028″ appliances with a 0.016″ × 0.022″ SS continuous archwire with a force of 200–300 g applied through a chain mesial to 34 and 44 above the center of resistance. The COP and AOP tipped between T1–T2–T3, while POP flattened between T1–T2–T3. All DMs decreased between T1–T2–T3 (Figure 3, Figure 4 and Figure 5; Table 1).

#### 3.2.3. Case 3

**Figure 6:**
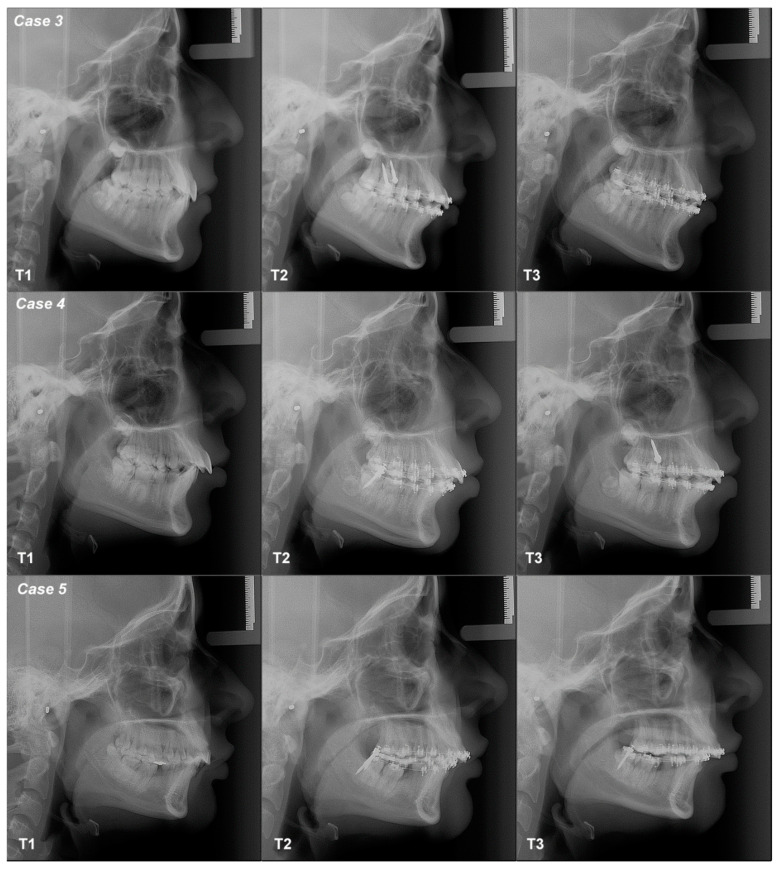
Class II patients digital lateral cephalograms.

A 13-year-old female patient with Class II canine relationships and skeletal Class II neutral angle. Her objective was to obtain Class I canine relations with two ***MS*** inserted at the infrazygomatic crest (IZC), achieved in eight months. The biomechanics included Roth 0.022″ × 0.028″ appliances with a 0.016″ × 0.016″ SS continuous archwire with a force of 200–300 grs applied through a chain mesial to 13 and 23, below the center of resistance. The COP, AOP and POP flattened from T1–T2, but they tipped between T2–T3. DMs for incisors and second molar increase between T1–T2 but decrease between T2–T3; first molar inclination decreases between T1–T2–T3. Third molars were present during all treatment observation periods (Figure 3, Figure 4 and Figure 6; Table 1).

#### 3.2.4. Case 4

A 13-year-old female patient with a Class II canine relationship and skeletal Class II low angle. Her objective was to verticalize the incisors with two MS at the BS, achieved after three months. The biomechanics included Roth 0.022″ × 0.028″ appliances with a 0.016″ × 0.016″ SS continuous archwire with a force of 200–300 g applied through a chain mesial to 34 and 44 above the center of resistance. The COP, AOP and POP flattened between T1–T2 but tipped between T2–T3. All DMs increased between T1–T2 but decreased between T2–T3. Third molars were present during all treatment observation periods (Figure 3, Figure 4 and Figure 6; Table 1).

#### 3.2.5. Case 5

A 31-year-old female patient with a Class II canine relationship and skeletal Class II low angle. Her objective was to verticalize the incisors with two MS at the BS, achieved after three months. The biomechanics included Roth 0.022″ × 0.028″ appliances with a 0.016″ × 0.022″ NiTi continuous archwire with a force of 200–300 grs applied through a chain mesial to 34 and 44, above the center of resistance. The COP and AOP tipped from T1–T2, but they flattened between T2–T3; POP flattened from T1–T2, but it tipped between T2–T3. DMs for incisors increased from T1–T2 and decreased between T2–T3; the first molar decreased between T1–T2–T3; the second molar decreased from T1–T2 and increased between T2–T3 (Figure 3, Figure 4 and Figure 6; Table 1).

#### 3.2.6. Case 6

**Figure 7:**
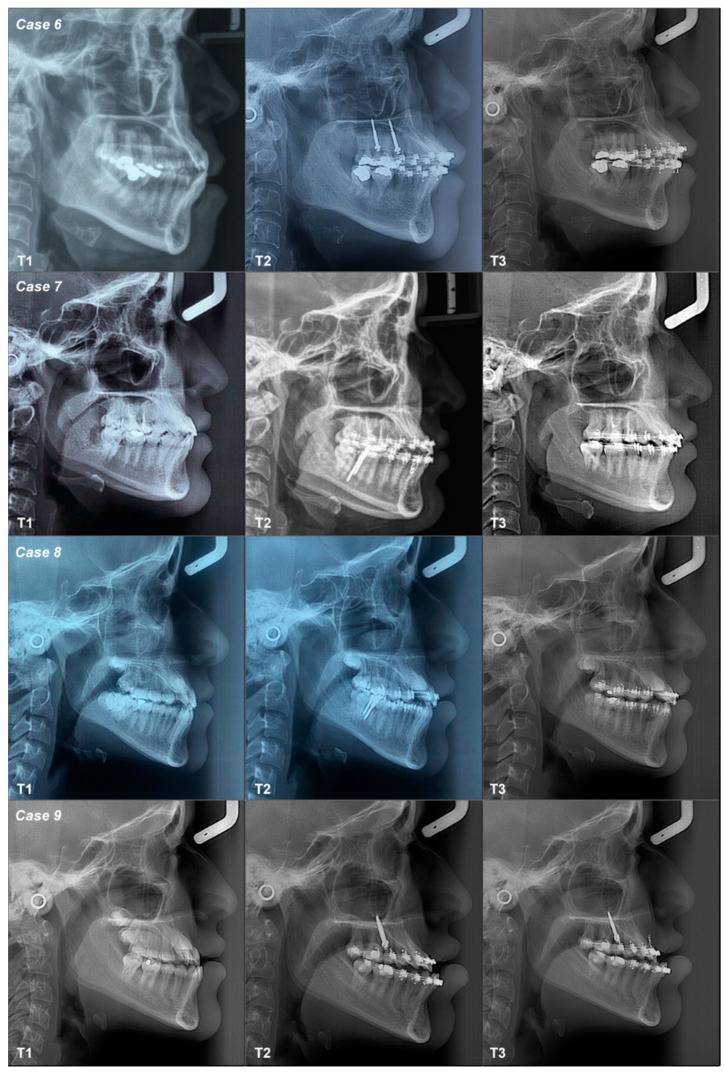
Class III patients digital lateral cephalograms.

A 54-year-old female patient with a Class I canine relationship and skeletal Class III high angle. Her objective was to verticalize the incisors with two MS inserted at the IZC, which was achieved in two months. The biomechanics included self-ligating 0.022″ × 0.028″ appliances with a 0.018″ × 0.025″ CuNiTi continuous archwire with a force of 200–300 grs applied through a chain mesial to 13 and 23 below the center of resistance. COP and AOP flattened between T1–T2–T3; POP flattened between T1–T2 but tipped from T2–T3. DM indicates that incisors increase between T1–T2 and decrease between T2–T3; first molar decrease between T1–T2 but increase between T2–T3; second molar decrease between T1–T2–T3 (Figure 3, Figure 4 and Figure 7; Table 1).

#### 3.2.7. Case 7

A 24-year-old female patient with Class I canine relationships and skeletal Class III high angle. Her objective was to verticalize their lower incisors with two MS at the BS, achieved after six months. The biomechanics included Roth 0.022″ × 0.028″ appliances with a 0.016″ × 0.022″ SS continuous archwire with a force of 200–300 grs applied through a chain to a hook distal to 33 and 43, parallel to the center of resistance. The COP and AOP flattened from T1–T2, but they tipped between T2–T3; POP tipped from T1–T2 but flattened between T2–T3. DMs, with respect to incisors and the second molar, decreased between T1–T2–T3, while the first molar increased from T1–T2 but decreased between T2–T3 (Figure 3, Figure 4 and Figure 7; Table 1).

#### 3.2.8. Case 8

A 13-year-old female patient with Class III canine relationships and skeletal Class III high angle. Her objective was to obtain Class I molar and canine relationships with two MS at the BS, achieved in six months. The biomechanics included Roth 0.018″ × 0.025″ appliances with a 0.016″ SS segmented archwire with a force less than 200 g applied through a chain mesial to 34 and 44, above the center of resistance. The COP tipped between T1–T2 but flattened between T2–T3; AOP flattened between T1–T2 but tipped between T2–T3; POP flattened between T1–T2–T3. DM measurements indicate that the incisors decreased from T1–T2 but increased between T2–T3; the first molar increased from T1–T2 but decreased between T2–T3; the second molar increased between T1–T2–T3 (Figure 3, Figure 4 and Figure 7; Table 1).

#### 3.2.9. Case 9

A 13-year-old female patient with Class III canine relationships and skeletal Class III high angle. Her objective was to increase sagittal discrepancy prior to orthognathic surgery with two MS at the IZC, achieved in four months. The biomechanics included self-ligating 0.022″ × 0.028″ appliances with a 0.017″ × 0.025″ SS continuous archwire with a force of 200–300 grs applied through a chain mesial to 13 and 23, below the center of resistance. The COP tipped from T1–T2 but flattened between T2–T3; AOP tipped between T1–T2–T3; POP flattened between T1–T2–T3. DMs, with respect to the incisors, decreased between T1–T2–T3; the first molar increased between T1–T2 but decreased between T2–T3; the second molar increased between T1–T2 but decreased between T2–T3 (Figure 3, Figure 4 and Figure 7; Table 1).

## 4. Discussion

MSs are very popular in orthodontics because they can be used in a high diversity of biomechanics [5,6]. One of these applications is the distalization of the entire arch to avoid dental extractions in camouflage treatments or to obtain better dentoalveolar positions of incisors and molars [15,16,17]; when this distal en-masse movement occurs, the OP could be affected [8,18,19] and, consequently, the dynamic of malocclusion and the sagittal position of the mandible could change [1]. In this case series, we tried to determine if there were changes in the OP, mandibular position and dentoalveolar measurements of incisors and molars through cephalometric analysis in patients of the orthodontics routine practice where the clinician determined to use MSs for obtaining distalization of the dental arches.

Our sample consisted of nine patients, all females; most of the studies related to temporary anchorage devices have a high prevalence of female patients (84.4%), which is according to our report [20]. The average age was 23.33 years old, but it must be considered that four of our patients were 13 years old at the beginning of orthodontic treatment; therefore, we still need to consider the influence of skeletal maturity and residual growth that could take place in the results [21].

In addition to the growth factors, the OP and dentoalveolar compensations may be affected by the orthodontic biomechanics variables more than the MS itself. The first phase of the treatment is the alignment and leveling of the teeth, which depends on the molar tip and vertical control of incisors and molars [22,23]. This can be correlated with our findings, where the variations between T1–T2 in OP and DM can be associated with factors based on vertical control, such as the positioning of the brackets and the expression of the arches.

During the retraction of teeth using biomechanics with MS, it is suggested that there is a rotation of the maxilla or mandible in a clockwise and counterclockwise direction, respectively, which occurs because the line of action of the force is occlusal to the center of resistance of the dental arch, which causes a dentoalveolar compensation with extrusion of the incisors and intrusion and distal inclination of the molars [24]. This can be related to the results between T1–T3 and T2–T3, where the changes in the inclination of the OP, the inclination of the incisors and the axial axes of the molars were evident; in most of the cases, no matter the skeletal pattern or the distalization arch, COP and AOP tipped because the retroinclination of the incisors, while POP flattened because the verticalization of the molars, and, the opposite arch, accompany the movements. Nevertheless, in Class II patients and in one of the Class III patients where the MSs were allocated in the maxilla, even the COP measurements report its flattening, the POP between T2–T3 tipped, which can be associated with the fact that a force applied below the center of resistance in the maxilla, produces a counterclockwise rotation of the OP [18,25].

According to the studies by Coro et al. [25] and Čelar et al. [26], there are significant differences in POP between the different skeletal malocclusions, with significant correlations with the mandibular position in the three planes of space: Class I and Class III patients have more flattened POP, while Class II patients have a steeper POP, findings corroborated in our study. After applying the biomechanics of distalization with MS, flattening of the POP could be beneficial for Class II patients but not for Class III patients because it can cause a counterclockwise rotation of the mandible, worsening their profile and dental relations.

Our study did not find any association between the SNA, SNB and ANB angle and the OP inclination, and it is consistent with other authors who reported factors such as the inclination of the cranial base and the upper jaw that could influence this measurement [27]. Also, according to Sato, OP can change the sagittal and vertical relation of the maxilla and mandible, changing the skeletal diagnosis [1]; nevertheless, even with the OP change, we did not find big modifications in the measurements of Kim analysis (APDI and ODI). This could explain because the objective of the orthodontic treatment of the patients was not to control the OP who considered other aspects like the absence of third molars. Sato suggests that third molars influence the position of the first and second molars through the squeezing out phenomenon and worsening the OP inclination [1]. In our study, cases 3 and 4 have their third molars present during all treatments, which can create resistance to second molar movement, evident in the DM of these patients between T1–T2. It can be presumed that MS distalization biomechanics has a dentoalveolar effect that can improve the occlusal stability and achieve a Class I relation, but this does not alter the antero-posterior or vertical relations of the mandible.

From a clinical perspective, the biomechanics of distalization with MS could be used with the aim of changing the inclination of the OP and thus facilitating the adaptation of the mandible to a more functional position [24,28]. Even this case series elucidates a little more about how the OP could behave with the biomechanics with MS; we recommended caution regarding the results because of the great heterogenicity of the sample, like the sociodemographic condition of the patients, skeletal patterns, clinician treatment objectives, and orthodontic biomechanics. Studies like clinical controlled trials with more homogeneous samples are required, to supervise thoroughly all the variables that can be related to the changes in the OP. Also, we suggest making studies that evaluate the clinical soft tissue profile, since it is an important factor to consider in the aesthetic result of the patient.

It is important to mention the strengths and limitations of this study. Although it is the first clinical approach to the phenomenon of interest, the nature of the design does not allow for the establishment of causal relationships. It is important to consider that conducting a randomized clinical trial is very difficult due to potential costs, patient recruitment, and the standardization of treatments. For this reason, the research group chose to analyze cases conducted as part of the daily routine in private practices and the orthodontic clinic at the University of Antioquia. Therefore, it is important to reflect on and emphasize the variability among the different cases analyzed. The heterogeneity of the patients does not allow for generalization of the results. However, despite these limitations, this study can provide insight into the routine reality of clinical practice for orthodontists.

## 5. Conclusions

Considering the above limitations, this study allows us to describe diverse changes when the distalization of the maxillary/mandibular arch was assisted by mini-screws (MS). In general terms, in the study sample, this conventional orthodontic treatment modifies the OP during the first phases of treatment. Several specific considerations should be considered, such as

- Distalization mechanics with ***MS*** steep the ***COP*** and the ***AOP*** but flatten the ***POP***, which can be positive for managing patients with a class II skeletal pattern;
- The anteroposterior position of the maxilla and the mandible, and the sagittal and vertical relationships, do not change with the biomechanics of distalization with ***MS***;
- The changes in the OP were associated with dentoalveolar inclination of incisors and molars;
- New methodological approaches should be considered, such as the use of clinical trials with statistical power and the incorporation of new variables and control groups.

## Figures and Tables

**Table 1:** Behavior of the occlusal plane, dentoalveolar measurements and mandibular position.

Case	Skeletal Diagnosis	Distalization Arch	Time	COP	AOP	POP	I	1M	2M	SMP	VMP
1	Class I Neutral Angle	Mandible	T1–T2	Flattened	Tipped	Flattened	Decreased	Increased	Decreased	Not changed	Not changed
			T2–T3	Tipped	Tipped	Flattened	Decreased	Decreased	Decreased	Not changed	Not changed
			T1–T3	Flattened	Tipped	Flattened	Decreased	Decreased	Decreased	Not changed	Not changed
2	Class I Low Angle	Mandible	T1–T2	Tipped	Tipped	Flattened	Decreased	Decreased	Decreased	Not changed	Not changed
			T2–T3	Tipped	Tipped	Flattened	Decreased	Decreased	Decreased	Not changed	Not changed
			T1–T3	Tipped	Tipped	Flattened	Decreased	Decreased	Decreased	Not changed	Not changed
3	Class II Neutral Angle	Maxilla	T1–T2	Flattened	Flattened	Flattened	Increased	Decreased	Increased	Not changed	Not changed
			T2–T3	Tipped	Tipped	Tipped	Decreased	Decreased	Decreased	Changed	Not changed
			T1–T3	Tipped	Tipped	Flattened	Decreased	Decreased	Decreased	Changed	Not changed
4	Class II Low Angle	Mandible	T1–T2	Flattened	Flattened	Flattened	Increased	Increased	Increased	Not changed	Not changed
			T2–T3	Tipped	Tipped	Tipped	Decreased	Decreased	Decreased	Not changed	Not changed
			T1–T3	Tipped	Flattened	Flattened	Increased	Decreased	Decreased	Not changed	Not changed
5	Class II Low Angle	Mandible	T1–T2	Tipped	Tipped	Flattened	Increased	Decreased	Decreased	Not changed	Not changed
			T2–T3	Flattened	Flattened	Tipped	Decreased	Decreased	Increased	Not changed	Not changed
			T1–T3	Tipped	Tipped	Flattened	Decreased	Decreased	Decreased	Not changed	Not changed
6	Class III High Angle	Maxilla	T1–T2	Flattened	Flattened	Flattened	Increased	Decreased	Decreased	Not changed	Not changed
			T2–T3	Flattened	Flattened	Tipped	Decreased	Increased	Decreased	Not changed	Not changed
			T1–T3	Flattened	Flattened	Flattened	Decreased	Decreased	Increased	Not changed	Not changed
7	Class III High Angle	Mandible	T1–T2	Flattened	Flattened	Tipped	Decreased	Decreased	Decreased	Not changed	Not changed
			T2–T3	Tipped	Tipped	Flattened	Decreased	Increased	Decreased	Not changed	Not changed
			T1–T3	Flattened	Tipped	Flattened	Decreased	Decreased	Decreased	Not changed	Not changed
8	Class III High Angle	Mandible	T1–T2	Tipped	Flattened	Flattened	Decreased	Increased	Increased	Not changed	Not changed
			T2–T3	Flattened	Tipped	Flattened	Increased	Decreased	Increased	Not changed	Not changed
			T1–T3	Tipped	Flattened	Flattened	Decreased	Decreased	Decreased	Not changed	Not changed
9	Class III High Angle	Maxilla	T1–T2	Tipped	Tipped	Flattened	Decreased	Increased	Increased	Not changed	Not changed
			T2–T3	Flattened	Tipped	Flattened	Decreased	Decreased	Decreased	Not changed	Not changed
			T1–T3	Tipped	Tipped	Flattened	Decreased	Increased	Increased	Not changed	Not changed

COP: Conventional occlusal plane. AOP: Anterior occlusal plane. POP: Posterior occlusal plane. I: Incisor. 1M: First molar. 2M: Second molar. SMP: Sagittal mandibular position. VMP: Vertical mandibular position.

## Data Availability

Data are unavailable due to privacy or ethical restrictions.

## Supplementary Materials

The following supporting information can be downloaded at https://www.mdpi.com/article/10.3390/dj12090278/s1, Table S1: Cephalometric analysis; Table S2: Sociodemographic, clinical and biomechanical variables.
