# Supplementary material for: Occlusal Plane, Mandibular Position and Dentoalveolar Changes during the Orthodontic Treatment with the Use of Mini-Screws
Source: Dent J (Basel). 2024 Aug 30;12(9):278. doi: 10.3390/dj12090278 (PMC11431644; doi:10.3390/dj12090278)
Supplement: Supplementary file 1 [file dentistry-12-00278-s001.zip › dentistry-3070189-supplementary.pdf]

Supplementary Table I. Cephalometric analysis.

Variables	Description
**Cephalometric points**	
Sella (S)	Point located in the center of the sella turcica
Nasion (N)	Point of union of the frontal bone and the nasal bones
Porion (Po)	Most posterosuperior point of the external auditory canal
Orbitale (Or)	Lowest point of orbital rim
A point (A)	Deepest point of the anterior concavity of the maxillary alveolar bone
B point (B)	Deepest point of the anterior contour of the mandibular symphysis
Anterior nasal spine (ANS)	Point corresponding to the spinous process of the maxilla
Posterior nasal spine (PNS)	Point of the most posterior projection of the junction of the palatal bones
Gonion (Go)	Point constructed by bisecting the angle formed by the posterior surface of the ramus and the mandibular body
Menton (Mn)	Point of the lower limit of the mandibular symphysis curve
Pogonion (Pog)	Most prominent point of the mandibular symphysis
Maxillary/Mandibular central incisor (MCI)	Incisal edge of the central incisor
Maxillary/Mandibular second premolar (MSP)	Cusp tip of the second premolar
Maxillary/Mandibular first molar (MM1)	Midpoint of the first molar
Maxillary/Mandibular second molar or third molar (MM2)	Midpoint of the second molar or third molar (if it has erupted) at the occlusal surface
**Cephalometric planes**	
Frankfurt horizontal plane (FH)	Formed by the union of Po and Or points
Palatal plane (PP)	Formed by the union of ANS and PNS points
Mandibular plane (MP)	Formed by the union of Go and Mn points
AB plane (AB)	Formed by the union A and B points
Facial plane (NPog)	Formed by the union of N and Pog points
Anterior cranial base plane (SN)	Formed by the union of S and N points
NA plane (NA)	Formed by the union of N and A points
NB plane (NB)	Formed by the union of N and B points
Conventional occlusal plane (COP)	Formed by the union of MCI to MM1
Anterior occlusal plane (AOP)	Formed by the union of MCI to MSP
Posterior occlusal plane (POP)	Formed by the union of MSP to MM2
⊥ FH-PP	Formed by a perpendicular line of FH from PP
Incisor axis (IMx-IMd)	Formed by a line crossing the longitudinal axis of the central incisors from the incisal edge to the apical to the root apex, both maxilla and mandible
First molar axis (1MMx-1MMd)	Formed by a line crossing the longitudinal axis of the first molar from the occlusal edge of the mesial cusp to the mesial root apex, both maxilla and mandible
Second molar axis (2MMx-2MMd)	Formed by a line crossing the longitudinal axis of the second molar from the occlusal edge of the

	mesial cusp to the mesial root apex, both maxilla and mandible
**Cephalometric angles**	
FH-MP	Angle formed by FH and MP
FH-PP	Angle formed by FH and PP
FH-NPog	Angle formed by FH and facial plane
AB-NPog	Angle formed by AB and facial plane
AB-MP	Angle formed by AB plane and MP
PP-MP	Angle formed by PP and MP
FH-COP	Angle between the FH plane and COP
FH-AOP	Angle between the FH plane and AOP
FH-POP	Angle between the FH plane and POP
Mx-COP	Angle of COP to the ⊥ FH-PP
Mx-AOP	Angle of AOP to the ⊥ FH-PP
Mx-POP	Angle of POP to the ⊥ FH-PP
OP Difference (A – P)	Angle between AOP and POP
Incisor axis	Angle between IMx-IMd and MP
Molar axis	Angle between 1MMx-2MMx-1MMd-2MMd and MP

Variables	Description	Mean
**Kim analysis**		
APDI	The resultant reading obtained from the arithmetic sum of FH-NPog angle, AB-NPog angle and FH-PP angle	Class I: 81.4° ± 3.7<br>Class II: 77.7 or less<br>Class III: 85.1 or more
ODI	The resultant reading obtained from the arithmetic sum of AB-MP angle and FH-PP angle	Neutral angle: 74.5° ± 6.07°<br>High angle: 68.4° or less<br>Low angle: 80.6° or more
**Riedel analysis**		
SNA	Angle formed by SN and NA planes	82.01 ± 3.89
SNB	Angle formed by SN and NB planes	79.97 ± 3.69
ANB	Angle between NA and NB planes	2.04 ± 1.81

Supplementary Table II. Sociodemographic, clinical and biomechanical variables.

Variables	Sample (n=9)	
	n	%
**Sociodemographic**		
Sex		
Males	0	0.0
Females	9	100.0
Age		
≤ 20	4	44.5
≥ 20	5	55.5
**Clinical/Skeletal**		
Molar relationships		
Class I	3	33.3
Class II	3	33.3
Class III	3	33.3
Canine relationships		
Class I	2	22.25
Class II	5	55.5
Class III	2	22.25
Sagittal skeletal pattern		
Class I	2	22.2
Class II	3	33.3
Class III	4	44.5
Vertical skeletal pattern		
Neutral	2	22.2
High angle	4	44.5
Low angle	3	33.3
Presence of third molars in the distalization arch		
No third molars at the beginning of treatment	3	33.3
Extraction of third molars between T1-T2	4	44.5
Presence of third molars during all treatment	2	22.2
**Biomechanical**		
Prescription		
Roth	7	77.7
Self-ligating	2	22.3
Material		
Stainless steal	9	100.0
Other	0	0.0
Slot size		
0.018″x0.025″	1	11.2
0.022″x0.028″	8	88.8
Mini-screws diameter/length (n=18)		
2x12mm	9	50.0
2x14mm	5	27.7
2x17mm	4	22.3
Mini-screws allocation		

Infrazygomatic crest	3	33.4
Mandibular buccal shelf	6	66.6
Distalization biomechanics		
Elastic chain to the arch mesial to canines	3	33.3
Elastic chain to the arch mesial to first premolars	5	55.5
Elastic chain to a hook mesial to first premolars	1	11.2
Direction of the force		
Parallel to the center of resistance of maxilla/mandible	1	11.2
Higher to the center of resistance of maxilla/mandible	5	55.5
Below to the center of resistance of maxilla/mandible	3	33.3
Force applied (grs)		
≤ 200	1	11.2
200-300	7	77.6
300-400	1	11.2
≥ 400	0	0.0
Reason to stop distalization		
To obtain class I canine relationship	2	22.3
To see clinically the incisors in a better vertical position	6	66.55
To see the sagittal discrepancy augmentation	1	11.15
Total time of distalization		
≤ 3 months	2	22.25
3-6 months	5	55.5
≥ 6 months	2	22.25
Distalization of the opposite arch to improve overjet		
Yes	2	22.2
No	7	77.8
**Total**	**9**	**100.0**
